# Assessment of macrophyte, heavy metal, and nutrient concentrations in the water of the Nairobi River, Kenya

**DOI:** 10.1007/s10661-017-6159-0

**Published:** 2017-08-16

**Authors:** Samwel Maina Njuguna, Xue Yan, Robert Wahiti Gituru, Qingfeng Wang, Jun Wang

**Affiliations:** 1 Sino-Africa Joint Research Center, Chinese Academy of Sciences, Wuhan, 430074 China; 2 University of Chinese Academy of Sciences, Beijing, 100049 China; 3 Key Laboratory of Aquatic Botany and Watershed Ecology, Wuhan Botanical Garden, Chinese Academy of Sciences, Wuhan, 430074 China; 4 Jomo Kenyatta University of Agriculture and technology, Juja, Kenya

**Keywords:** Nairobi River, Heavy metals, Nutrients, Macrophytes

## Abstract

Nairobi River tributaries are the main source of the Athi River. The Athi River basin is the fourth largest and important drainage system in Kenya covering 650 km and with a drainage area of 70,000 km^2^. Its water is used downstream by about four million people not only for irrigation but also for domestic purposes. However, its industrial, raw sewer, and agricultural pollution is alarming. In order to understand distribution and concentration of heavy metals and nutrients in the water of Nairobi River, 28 water samples were collected in the rainy season (October) of 2015 and dry season (June) of 2016. Cd, Cu, Cr, Zn, As, Pb, Fe, Ni, Mn, NO_3_^−^, and TP were analyzed. Only Cr, Pb, Fe, and Mn had concentrations exceeding the WHO permissible limit for drinking water. Out of the 28 sites examined in the study, one site had Pb exceeding the WHO recommended level. Similarly, three sites exceeded the same level for Cr. Only three sites were within the WHO permissible limits for drinking water for Mn while just four sites were within USEPA limit for Fe. Industrial effluent, domestic sewerage, agricultural activities, and solid waste were the main sources of pollution. Significant spatial variation of both heavy metals and nutrients concentration was observed and emanated from point source pollution. Eleven out of 31 macrophytes species that were identified along the river and its tributaries are effective heavy metal and nutrient bioaccumulators and may be used in phytoremediation.

## Introduction

Heavy metal pollution in aquatic ecosystems has become a global concern (Kifayatullah et al. 2013). Bioaccumulation, toxicity, and persistence in the environment aggravate heavy metal hazardousness (Enderlein et al. 1996). Pollution can be a result of either geological or anthropogenic processes. Heavy metals released due to geological processes such as rock weathering and volcanic eruptions are discharged into water bodies via run off, erosion, and floods. Anthropogenic activities such as leaching of fertilizers, improper industrial effluent disposal, accidental oil spillage, domestic sewerage, minerals mining, and rain water contaminated with heavy metals in the atmosphere are thought to significantly contribute to aquatic ecosystem pollution (Ferati et al. 2015; Kifayatullah et al. 2013; Varol and Şen 2012).

Heavy metals such as Hg, Cd, Cr, As, Pb, and Mn are increasingly getting associated with kidney problems, neurological and cancer ailments, and in some cases vital nutrient deficiencies inhibiting proper functioning of the human immune system (Cobbina et al. 2015; Water Research Australia 2013). Arsenic has particularly been linked to abortion, still births, and cardiovascular problems (WHO 2010). In a study conducted in Bangladesh, students whose drinking water had a high concentration of Mn got 6.4 points lower in mathematics compared to children whose water contained little or no manganese (Khan et al. 2012).

Past studies have recorded rivers with heavy metals greater than the WHO standards for drinking water. For instance, 0.89 mg/L Mn in Asunle River in Nigeria (Ogunfowokan et al. 2013), 781 μg/L Pb in Tibetan Rivers (Huang et al. 2008), 0.2 mg/L Ni in Khoshk River in Iran (Salati and Moore 2010), and 2.297 mg/L Cr and 1.051 mg/L Pb in Challawa River in Nigeria (Dan’azumi and Bichi 2006).

Utilizing 3% of the total earth fresh water of which only less than 1% is usable might become a challenge in the future (Pegram 2010). Food production, industrial output, and domestic water needs are bound to increase to satisfy a larger population that is projected to reach 9.2 billion in 2050 (Krhoda 2006; UN 2007).

Kenya is classified as a water-scarce country with a water supply of 690m^3^ per capita per annum against the global benchmark of 1000 m^3^ (Birongo and Le 2005). The country has been experiencing rapid population growth from 10.9 million in 1969 to a projected 65.9 million in 2030 (Population Reference Bureau 2011). In order to meet increasing domestic, agricultural, and industrial water needs, prudent use and conservation of available water resources is paramount. However, the Nairobi River basin, which is part of the Athi River basin and the fourth largest and important drainage system in Kenya covering 650 km, was reported to be the most polluted in Kenya in 2009 (Baseline Report 2012; UNEP/Nairobi & Wiomsa 2009). The Athi River traverses arid areas in eastern and coastal regions, and its water is used for livestock, irrigation and domestic purposes (Muiruri et al. 2013).

In order to address Nairobi River’s pollution problem, it is critical to understand its pollutants, their concentration, and even the point and non-point sources of pollution. A previous documented study on heavy metal contamination of Nairobi River water was conducted 10 years ago. This is quite a long time considering Nairobi’s rapid population growth and industrial production dynamics. It may also have failed to capture comprehensively spatial pollution, which is vital in exploring pollution source, since the study had just six sampling sites.

In this study, water samples were collected from Nairobi River in both dry and wet seasons. Heavy metal and nutrient concentration in a river may be influenced by weather pattern. Wet weather may dilute pollutant concentration while hot and dry weather may result in a high concentration. Macrophytes at designated areas along the river were also identified to investigate Nairobi River self-purification mechanism.

The objectives of this study were (1) to estimate the concentration of heavy metals and nutrients in the water of Nairobi River, (2) explore potential pollution sources of heavy metals and nutrients, and (3) evaluate the Nairobi River self-purification mechanism.

## Materials and method

### Study area

Nairobi River is the main tributary of the Athi River. The Athi River basin is the fourth largest and important drainage system in Kenya covering 650 km and with a drainage area of 70,000 km^2^ (Kithiia 1997). It traverses seven counties, Kiambu, Nairobi, Machakos, Kitui, Taita Taveta, Tana River, and Kilifi before draining its water into the Indian Ocean at Malindi (Krhoda 2006). Its water is used downstream by about 4 million people for irrigation and domestic purposes (Musyoki et al. 2013).

Nairobi River has three main tributaries, Mathare, Nairobi, and Ngong Rivers. The river passes through Nairobi, a city with a population of approximately 3.5 million people. Its three tributaries traverse numerous informal settlements such as Mathare valley, Korogocho, Majengo, Dandora, and Kariobangi South. These areas are inhabited by underprivileged people with poor access to a sewerage and solid waste disposal system. This coupled with the presence of poorly regulated light industries along the course of the river aggravate pollution (Musyoki et al. 2013). Additionally, Nairobi River tributaries are surrounded by small-scale farms producing fresh vegetables (Karanja et al. 2010). Riparian vegetation along these tributaries, that is vital in curbing erosion and natural water remediation, has been extensively cleared for farming

### Sampling sites

**Fig. 1:**
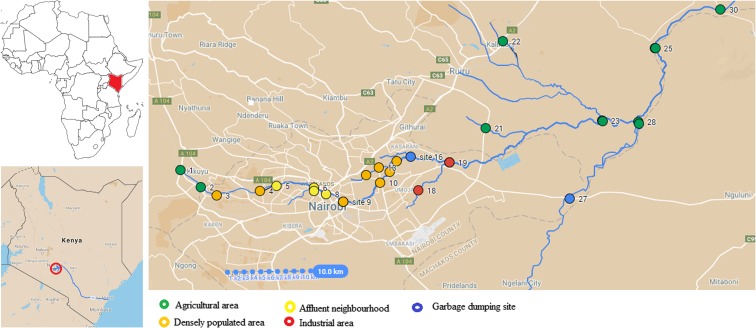
Map of water sampling and macrophytes identification sites along Nairobi River

Sampling site selection was based on land use pattern, economic activities, population demographics, and suspected pollutant point sources. Twenty-eight water sampling and 29 macrophyte identification sites were selected as detailed in Fig. 1. The first site, the source of Nairobi River, was the Ondiri swamp in Kikuyu. The swamp is about 100 m from the Southern By-pass highway and about 1.5 km from Kikuyu town at an elevation of 2007 m above sea level and with an approximate area of 0.468 Km^2^. Its average annual rainfall is 800–1200 mm of which 80% is experienced between March and May, while the remaining 20% falls between September and October. Soils at the Ondiri swamp are mostly kaolinite with plenty of humus on the surface. From the swamp, the river flows through agricultural land at Kikuyu and Dagoreti before getting to densely populated areas at Kawangware, Chiromo campus, Globe roundabout, Gikomba, Kariobangi South, traverses Njiru plains, flows past Fourteen falls in Machakos county before joining Athi and eventually the Sabaki River which drains its water into the Indian Ocean at Malindi in Kenya (Awuor 2008; Budambula and Mwachiro 2006).

### Sample collection

Water samples were collected twice. During the rainy season (October) of 2015 and dry season (June) of 2016. Since the Nairobi River is homogenous, with shallow water averaging 0.3–1.5 m deep and with a steady flow from the source to Fourteen falls, where the last sample was collected, river water vertical change was insignificant. Samples of 500 mL water were collected below river water using pre-cleaned polyethylene bottles. The bottles were filled completely to exclude any air present and capped immediately. Each sample was divided into two on arrival at the Kenya Bureau of Standards where analysis was conducted. Each sample of 250 mL was filtered through a 0.45-μm cellulose acetate membrane filter. Nitric acid was added to lower the pH to < 2 to avoid precipitation after which the samples were stored at 4 °C in a refrigerator prior to analysis (John 2000). Global positioning system (GPS) was used to mark sampling sites.

Macrophytes were identified at 29 designated sites and verified using Upland Kenya Wild Flowers (Agnew & Agnew 1994).

### Sample analysis

Concentration of dissolved target heavy metals was measured using inductively coupled mass spectrophotometer (ICP-MS) (Agilent, 7700 series). Argon gas, purity guaranteed at 99.99%, was used besides the external calibration approach since no sample-sample variability or interference effects were anticipated.

NO_3_^−^, P, and Fe concentration was determined using the spectrophotometric method (UV-1700 pharmaspec SHIMADZU) at an absorbance of 420, 880, and 515 nm, respectively, according to methods described in the reference (Frenesius et al. 1988; Bandtock and Hanson 1974; Method 365. 3 1978).

### Quality assurance and quality control

All reagents used were of high grade. Analytical quality was guaranteed by using analytical duplicates, blanks, calibration standards, and spike samples. To maximize accuracy and precision of results, calibration and check standards had to tally before sample analysis. Results were within certified values (Authority 2000; USEPA 1994).

### Statistical analysis

Pearson correlation analysis was done to determine interaction among heavy metals and nutrients. Principle component analysis, factor and cluster analysis were conducted to determine related and probable sources of pollution, either natural or anthropogenic. Statistical analysis was done using SPSS 20.0 (SPSS Inc.). Sampling sites map was generated from Google maps using global positioning system (GPS) coordinates.

## Results

### Heavy metal and nutrient concentration

Descriptive statistics of heavy metals and nutrients of Nairobi River are summarized in Table 1. Different sampling sites and seasons recorded varying concentrations of heavy metals and nutrients. For instance, Pb was first detected at the ninth sampling site downstream compared to Mn that was present in all sites and seasons apart from the Ondiri swamp in October. NO_3_^−^ and P were recorded in all sites in both seasons. However, their mean values were higher in June, when the weather was hot and dry, registering 30,936 and 1994 μg/L, compared to October when they were 487 and 1510 μg/L, respectively. All heavy metals and nutrients with exception of Cr, Fe, Mn, and Pb were within the WHO and USEPA permissible limits for drinking water. Cr, Fe, Mn, and Pb concentration ranged between 0–245, 0–11,900, 0–2915.03, and 0–158 μg/L, respectively.

**Table 1:** Concentrations of heavy metals and nutrients in waters of the Nairobi River (μg/L)

Heavy metals/nutrients	Dry season				Rainy season			
	Range	Mean	Std. deviation	Skewness	Range	Mean	Std. deviation	Skewness
Cr	0–245	33.14	66.01	2.3	0–4.75	1.47	0.94	1.92
Mn	0–2915.03	1003.21	634.93	0.97	0–2649	801.41	822.44	0.63
Ni	0–9	2.75	1.6	2.07	0–26.83	3.53	4.86	4.33
Cu	0–3	0.61	0.79	1.34	0–9.89	4.25	2.96	0.61
Zn	0–2568	104.86	482.84	5.29	0–154.22	32.27	37.19	1.57
As	0–1	0.18	0.39	1.78	0–4.00	1.74	1	0.01
Cd	0	0	0		0–0.46	0.12	0.13	1.37
Pb	0–158	5.89	29.82	5.29	0–9.00	1.17	2.49	2.3
NO_3_^−^	214–40,000	30,935.71	4506.88	0.37	15.4–3311.30	486.58	854.5	2.11
Fe	50–11,900	2496	2808	1.96	0–617.30	76.6	122.2	3.43
P	440–4370	1993.57	1244.09	0.53	472.3–2913.80	1509.91	650.74	0.91

Spatial concentration variation was conspicuous. Only one site, the Dandora garbage dumping site, exceeded the WHO permissible limit for Pb, recording 158 μg/L. Cr concentration was excessively high in three sites: in the Eastern By-pass highway site, Kalimoni and Chokaa area. Mn and Fe are the only metal elements that were prevalent in majority of the study sites. Only three sites were within the WHO permissible limits for drinking water for Mn while just four sites were within the USEPA limit for Fe.

Fe and Mn may have originated from both natural and anthropogenic sources; andisol soils prevalent in the study site are rich in Fe.

Cr, Pb, Zn, Cu, and Ni may have originated from industrial effluent and garbage with electronic waste. A high concentration of these metals was recorded downstream of Ngong River in the industrial area. For instance, Kayole, an area that is along the Ngong River, recorded the highest level of Zn, Pb, and As of 154.23, 4.5, and 3.98 μg/L, respectively, in the rain season. The present study is consistent with the previous study that showed of all the tributaries of the Nairobi River, the Ngong River was the most polluted (Nairobi River Basin-Baseline Report 2012).

### Interaction among heavy metals and nutrients

Pearson correlation analysis results are presented in Tables 2, 3 and 4 for the dry and rainy season, respectively. Correlation coefficient values for elements Cr, Mn, Fe, As, P, Cu, Ni, and Pb were positively correlated at (*p* < 0.01) in the dry season. Additionally, Cr, As, Pb, Mn, P, Ni, Cu, and Zn registered positive correlation in the rainy season at the same significance level. NO_3_^−^ had negative correlation with all heavy metals and P in both seasons. This could be linked to its agricultural source and its highly soluble nature. Heavy metals and nutrients that exhibited considerable positive correlation may have originated from a similar source, have identical properties, were influenced by related elements, or could be mutually dependent and vice versa (Makokha et al. 2015; Zhang et al. 2015). Principal component and cluster analysis were subsequently used to further explore sources of pollution.

**Table 2:** Nairobi River heavy metal concentration comparison with other rivers used as source of drinking water against WHO and USEPA guidelines (μg/L)

Rivers	Cd	Cr	Cu	Fe	Mn	Ni	Pb	Zn
Taipu River, China^a^	2	9	25	316	165	15	57	98
Yangtze River in Nanjing^b^	5	21	11	240	5	13	55	9
Tsurumi River, Japan^c^	–	217	**654**	362	264	**223**	339	–
Challawa River, Nigeria^d^	–	**924**	390	5668	1681	210	**840**	2227
Wusong River, China^e^	4	10	355	530	209	31	77	123
Lambro River, Italy^f^	0.1–4.8	66	1.1–134	–	–	–	2.2–138.8	–
Ruda River, Poland^g^	< 3	< 5	5–22	470–9610	179–1760	8–10	30–140	–
DilDeresi (stream), Turkey^h^	7	30	31	1310	–	–	80	220
Hindon River, India^i^	**12**	124	–	692	617	–	276	110
Nairobi River, Kenya (present study)	0.43	245	9.89	**11,900**	**2915**	26.83	158	**2568**
WHO drinking water guideline, 2008^j^	**3**	**50**	**2000**	–	**400**	**70**	**10**	**3000**
USEPA drinking water guideline, 2012^k^	**5**	**100**	**1300**	**300**	**1000**	–	**15**	**5000**

Rivers: en dash implies heavy metal not detected. WHO and USEPA: en dash implies guideline not given. Bold values: highest concentration recorded

^a, e^Hong et al., 2014

^b^Wu et al., 2009

^c^Mohiuddin et al., 2010

^d^Dan’azumi and Bichi, 2010

^f^Pettine et al., 1996

^g^Loska and Wiechula, 2003

^h^Pekey et al., 2004

^i^Suthar et al., 2009

^j^Gordon et al. 2008

^k^Bonnelle 1987

**Table 3:** Pearson correlation matrix of concentration among heavy metals and nutrients in water of the Nairobi River in the dry season, June

	Cr	Mn	Ni	Cu	Zn	As	Pb	Fe	NO_3_^−^	P
Cr	1									
Mn	**0.699** ^**a**^	1								
Ni	**0.574** ^**a**^	**0.448** ^**b**^	1							
Cu	0.235	**0.728** ^**a**^	0.154	1						
Zn	−0.072	0.199	0.034	0.361	1					
As	**0.415** ^**b**^	**0.741** ^**a**^	0.074	**0.721** ^**a**^	**0.426** ^**b**^	1				
Pb	−0.074	0.266	−0.089	**0.605** ^**a**^	−0.030	**0.425** ^**b**^	1			
Fe	**0.606** ^**a**^	**0.691** ^**a**^	0.087	**0.503** ^**a**^	−0.154	**0.450** ^**b**^	0.100	1		
NO_3_^−^	−0.019	−0.028	**–0.410** ^**b**^	−0.043	−0.275	−0.046	0.047	−0.278	1	
P	0.360	**0.687** ^**a**^	0.203	**0.594** ^**a**^	−0.013	**0.374** ^**b**^	0.226	**0.580** ^**a**^	0.081	1

Bold values represent correlation with significance

^a^Correlation is significant at 0.01 probability level

^b^Correlation is significant at 0.05 probability level

**Table 4:** Pearson correlation matrix of concentration among heavy metals and nutrients in water of the Nairobi River in the rainy season, October

	Cr	Mn	Ni	Cu	Zn	As	Pb	Fe	NO_3_^−^	P
Cr	1									
Mn	**0.421** ^**b**^	1								
Ni	0.125	0.202	1							
Cu	**0.376** ^**b**^	**0.430** ^**b**^	**0.561** ^**a**^	1						
Zn	**0.442** ^**b**^	**0.401** ^**b**^	**0.559** ^**a**^	**0.769** ^**a**^	1					
As	**0.665** ^**a**^	**0.632** ^**a**^	0.222	**0.636** ^**a**^	**0.508** ^**a**^	1				
Pb	**0.630** ^**a**^	0.318	0.125	**0.410** ^**b**^	**0.709** ^**a**^	**0.567** ^**a**^	1			
Fe	0.227	0.359	0.336	0.190	0.308	0.044	0.099	1		
NO_3_^−^	−0.200	**–0.491** ^**b**^	−0.274	**–0.472** ^**b**^	**–0.415** ^**b**^	−0.320	−0.166	−0.312	1	
P	**0.404** ^**b**^	**0.511** ^**a**^	0.186	**0.383** ^**b**^	**0.397** ^**b**^	**0.510** ^**a**^	0.372	0.337	**−0.414** ^**b**^	1

Bold values represent correlation with significance

^a^Correlation is significant at 0.01 probability level

^b^Correlation is significant at 0.05 probability level

### Probable sources of pollution

Principal component analysis (PCA) is a variable reducing technique. Consequently, it can be used to reduce the number of observed variables to a smaller number called principal component (PC). Principal components account for most of the observed variance. The number of principal components that are extracted during analysis is equal to the number of observed variables (Suhr 2005). Principal components are uncorrelated and the first few preserve most of the variation present in all the initial variables (Alonso Castillo et al. 2013). PCA may be applied to deduce probable sources of heavy metals which can either be natural or anthropogenic (Salati and Moore 2010). Varimax rotation was used in this study since it was assumed the variables were uncorrelated. Four principal components that explained 84.57% of total variance were extracted at Eigen value > 1, in the dry season. These results are comparable to other studies in Iran and China where component loadings for seven and eight variables were 82.78 and 81.64%, respectively (Salati and Moore 2010; Zhang et al. 2016).

Component one, two, three, and four explained 41.25, 17.83, 13.37, and 12.11% variance, respectively, as shown in Table 5. In component one, Fe, Cr, and Mn had the highest loading score. Pb and Cu had the highest positive loading of 0.90 and 0.74, respectively, in component two. NO_3_^−^ and Ni had significant positive loading score in component three of 0.86 and 0.79, while only Zn in component four had a high positive loading of 0.96.

**Table 5:** Rotational component matrix of heavy metals and nutrients in the Nairobi River water

Heavy metals and nutrients	Dry season				Rainy season		
	PC1	PC 2	PC 3	PC 4	PC 1	PC 2	PC 3
Fe	**0.88**					**0.70**	
Cr	**0.86**				**0.81**		
Mn	**0.85**	0.34			0.47	**0.70**	
P	0.65	0.43			0.46	0.64	
Pb		**0.90**			**0.83**		
Cu	0.46	**0.74**		0.37	0.44		**0.73**
As	0.48	0.53		0.52	**0.82**		
NO_3_^−^			**0.86**			**−0.69**	
Ni	0.41		**0.79**				**0.87**
Zn				**0.96**	0.52		**0.75**
Eigen value	4.13	1.78	1.34	1.21	4.53	1.39	1.17
variance %	41.25	17.83	13.37	12.11	45.27	13.90	11.72
Cumulative variance %	41.25	59.08	72.45	84.57	45.27	59.17	70.89

Bold loadings are > 6.6. Less than 0.3 loadings were not included

Further investigation of sources and relationship among heavy metals and nutrients was done using cluster analysis.

**Fig. 2:**
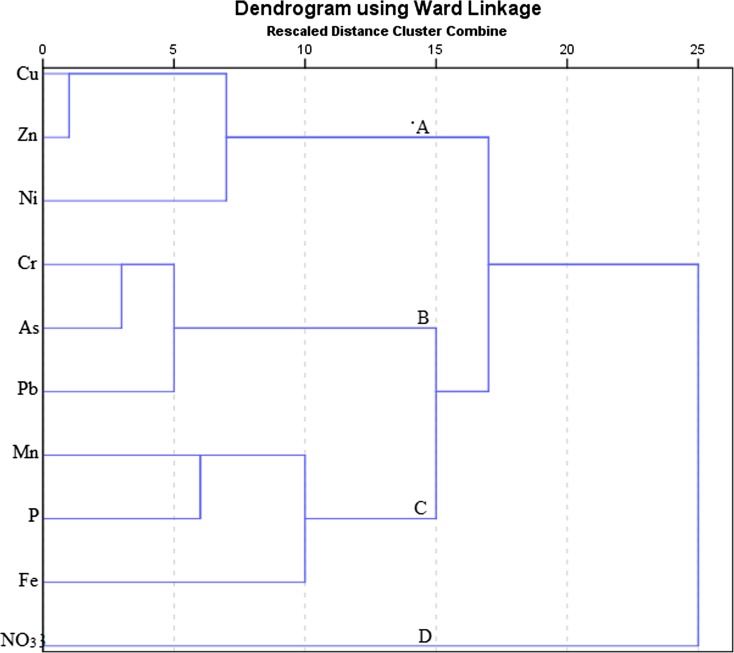
Hierarchical dendrogram of metal elements and nutrients in water of Nairobi River in the rainy season, October

**Fig. 3:**
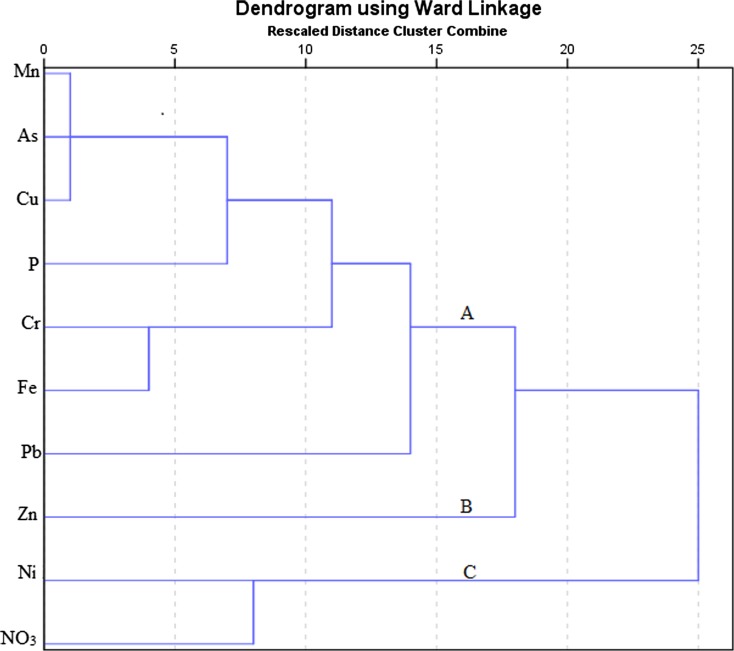
Hierarchical dendrogram of metal elements and nutrients in water of Nairobi River in the dry season, June

Cluster analysis is an exploratory data analysis tool that groups a set of variables that are closely related together and enhances understanding of relationships. In the present study, the hierarchical cluster analysis method was used (Figs 2 and 3). Euclidean interval pattern and ward linkage were applied while heavy metal and nutrient concentrations were standardized using Z-score.

There were four clusters in rainy season and three clusters with five distinct groups in the dry season dendrogram. Groups formed were categorized based on their element characteristics and sources. Cluster one in the rainy season dendrogram was composed of Cu, Zn, and Ni. These metals may have originated from garbage with electronic waste since the highest concentration was detected at Kayole where dumping was done directly into the river.

Pb contamination, probably from lead acid batteries, was detected near the Dandora dumping site in the dry season. Improper dumping of garbage has been identified as a significant source of pollution (Ideriah et al. 2012).

Zn was from garbage in the rainy season. Additional pollution may have originated from industrial effluent, industrial area, in the dry season.

Cr and Fe may have emanated purely from industrial effluent from the tannery at Chokaa in the dry season besides natural sources in the rainy season (Li and Zhang 2010; Mohiuddin et al. 2011). While Mn, As, and Cu pollution was from natural and industrial effluent (from Industrial area) in the dry season, Mn originated from natural sources and leaking sewerage in the rainy season. Mn may naturally be found in water due to rock weathering and soil erosion (Water Research Australia 2013). P may have been from natural sources, especially decaying organic matter, in both seasons.

Although some NO_3_^−^ may have been from leaking sewerage in the dry season, all NO_3_^−^ in the rainy season was due to leaching from farms in the cleared riparian zone.

### Relationship between macrophytes distribution and the Nairobi River self-purification

Macrophytes play an important role in aquatic ecosystems. They have been reported to remove, transform, or stabilize heavy metals in water and sediments (Tangahu et al. 2011). Macrophytes can uptake heavy metals either from sediment through roots or from water through leaves (Wang et al. 2014). Phytoextraction, phytostabilazation, phytostimulation, phytovolatalization, and rhizofiltration are the key mechanisms in macrophyte phytoremediation (Vardanyan et al. 2008).

Macrophytes growing along the Nairobi River were identified at 29 designated sites. Thirty-one plant species from 23 families were prevalent as displayed in Table 6. Eleven species of the identified macrophytes are heavy metal bioaccumulators. For instance, *Leersia hexandra* and *Pennisetum purpureum* have been found to effectively remove Cr in water (Study 2011; Liu et al. 2015). *Canna indica* was used in TN removal (Cui et al. 2010). *Colocasia* spp. and *Amaranthus* spp. were good at extracting Cd from soil, while *Tithonia diversifolia* was effective in uptaking Pb. (Adesodun et al. 2010; Mazumdar and Das 2015; Zhang et al. 2010a, 2010b )*. Eichhornia crassipes* was found to efficiently remove NO_3_^−^_,_ Fe, Zn, Cu, Cd, and Cr while *Cyperus rotundus* uptook Cd and Cr from contaminated water and soil, respectively (Petrucio and Esteves 2000; Sood et al. 2012; Swamy 2014). *Ricinus communis* was found to accumulate Cd, Pb, Ni, As, and Cu from contaminated soil (Bauddh et al. 2015). *Cyperus articulatus* removed As, Cd, Cr, Cu, Fe, Hg, Mn, Ni, and Pb from water (Farrag and Fawzy 2012) while *Typha domingensis* decontaminated Hg and Cr from water (Gomes et al. 2014; Sultana et al. 2014)*.*

**Table 6:** Dominant macrophytes in the Nairobi River water sampling points

Site	Species	Family
1	*Commelina benghalensis*	Commelinaceae
	*Cyperus articulatus*	Cyperaceae
	*Leersia hexandra*	Poaceae
	*Cyperus rotundus*	Cyperaceae
	*Typha domingensis*	Typhaceae
2	*Cardamine obliqua*	Brassicaceae
	*Pavonia urens*	Malvaceae
	*Polygonum senegalense*	Polygonaceae
	*Peucedanum scottianum*	Apiaceae
3	*Canna indica*	Cannaceae
	*Colocasia spp.*	Araceae
	*Cyperus distans*	Cyperaceae
	*Dryoathrium boryanum*	Athyriaceae
	*Polygonum senegalense*	Polygonaceae
	*Peucedanum scottianum*	Apiaceae
4	*Cardamine obliqua*	Brassicaceae
	*Commelina benghalensis*	Commelinaceae
5	*Canna indica*	Cannaceae
	*Commelina benghalensis*	Commelinaceae
	*Plantago africana*	Plantaginaceae
	*Polygonum senegalense*	Polygonaceae
6	*Amaranthus hybridus*	Amaranthaceae
	*Bidens pilosa*	Asteraceae
	*Commelina benghalensis*	Commelinaceae
7	*No macrophytes*	
8	*Commelina benghalensis*	Commelinaceae
	*Cyperus michelianus*	Cyperaceae
	*Cardamine obliqua*	Brassicaceae
	*Plantago africana*	Plantaginaceae
9	*Cyperus distans*	Cyperaceae
	*Amaranthus hybridus*	Amaranthaceae
	*Plantago africana*	Plantaginaceae
10	*Plantago africana*	Plantaginaceae
	*Amaranthus hybridus*	Amaranthaceae
	*Canna indica*	Cannaceae
	*Polygonum senegalense*	Polygonaceae
11	*Amaranthus hybridus*	Amaranthaceae
	*Polygonum senegalense*	Polygonaceae
	*Plantago africana*	Plantaginaceae
	*Typha domingensis*	Typhaceae
12	*Amaranthus hybridus*	Amaranthaceae
	*Polygonum senegalense*	Polygonaceae
	*Commelina benghalensis*	Commelinaceae
	*Pennisetum purpureum*	Poaceae
	*Pennisetum massaicun*	Poaceae
13	*Canna indica*	Cannaceae
	*Commelina benghalensis*	Commelinaceae
	*Plantago africana*	Plantaginaceae
	*Amaranthus hybridus*	Amaranthaceae
	*Polygonum senegalense*	Polygonaceae
	*Ipomoea spp.*	Convolvulaceae
14	*Commelina benghalensis*	Commelinaceae
	*Polygonum senegalense*	Polygonaceae
	*Tithonia diversifolia*	Asteraceae
15	*Amaranthus hybridus*	Amaranthaceae
	*Polygonum senegalense*	Polygonaceae
	*Pavonia urens*	Malvaceae
	*Cyperus michelianus*	Cyperaceae
16	*Colocasia spp.*	Araceae
	*Polygonum senegalense*	Polygonaceae
	*Tithonia diversifolia*	Asteraceae
17	*Polygonum senegalense*	Polygonaceae
	*Tithonia diversifolia*	Asteraceae
	*Colocasia spp.*	Araceae
18	*Polygonum senegalense*	Polygonaceae
	*Amaranthus hybridus*	Amaranthaceae
	*Commelina benghalensis*	Commelinaceae
19	*Polygonum senegalense*	Polygonaceae
	*Amaranthus hybridus*	Amaranthaceae
20	*Polygonum senegalense*	Polygonaceae
	*Amaranthus hybridus*	Amaranthaceae
	*Tithonia diversifolia*	Asteraceae
21	*Colocasia spp.*	Araceae
	*Eichhornia crassipes*	Pontederiaceae
	*Polygonum senegalense*	Polygonaceae
	*Commelina benghalensis*	Commelinaceae
22	*Pavonia urens*	Malvaceae
	*Cyperus schimperianus*	Cyperaceae
	*Colocasia spp.*	Araceae
	*Polygonum senegalense*	Polygonaceae
	*Pavonia urens*	Malvaceae
	*Typha domingensis*	Typhaceae
23	*Polygonum senegalense*	Polygonaceae
	*Amaranthus hybridus*	Amaranthaceae
	*Commelina benghalensis*	Commelinaceae
	*Cyperus schimperianus*	Cyperaceae
	*Colocasia spp.*	Araceae
24	*Datura stramonium*	Solanaceae
	*Amaranthus hybridus*	Amaranthaceae
	*Polygonum senegalense*	Polygonaceae
	*Ricinus communis*	Euphobiaceae
	*Solanum nigrum*	Solanaceae
	*Malva parvifolia*	Malvaceae
	*Tithonia diversifolia*	Asteraceae
25	*Eichhornia crassipes*	Pontederiaceae
	*Polygonum senegalense*	Polygonaceae
	*Commelina benghalensis*	Commelinaceae
	*Tithonia diversifolia*	Asteraceae
	*Basella alba*	Basellaceae
	*Achyranthes aspera*	Amaranthaceae
26	*Eichhornia crassipes*	Pontederiaceae
	*Amaranthus hybridus*	Amaranthaceae
	*Sphaeranthus gomphrenoides*	Asteraceae
28	*Polygonum senegalense*	Polygonaceae
	*Oxalis latifolia*	Oxalidaceae
	*Commelina benghalensis*	*Commelinaceae*
	*Cyperus articulatus*	Cyperaceae
29	*Gomphocarpus fruticosus*	Asclepiadoacea
	*Cyperus articulatus*	Cyperaceae
30	*Eichhornia crassipes*	Pontederiaceae
	*Colocasia spp.*	Araceae

## Discussion

Kenya has experienced rapid population growth since independence. High fertility and declining child mortality rates have accelerated population growth from 10 million at independence to the current 42 million (Thuku et al. 2013). Rural-urban migration as people move in search of employment and improved social services in urban areas has not experienced a corresponding infrastructure expansion. Sluggish economic growth coupled with limited availability of decent jobs has led to mushrooming of numerous informal settlements. These informal settlements have poor wastewater drainage systems and solid waste disposal mechanisms (Ngumba et al. 2016). The Nairobi River tributaries that traverse these informal settlements act as sewerage and garbage dumping sites (Amnesty International 2009).

Domestic wastewater discharged directly into the river may have contaminated the Nairobi River water with Mn from potassium permanganate that is used as a household disinfectant.

Previous study of the Nairobi River recorded high levels of Pb, Mn, and Fe, exceeding the WHO drinking water recommended rate (Budambula and Mwachiro 2006). This is consistent with the current study where Pb, Mn, and Fe were equally above the WHO recommended level at some sampling points. Pb pollution may have been from electronic waste from the Dandora dumping site and effluent from the industrial area (Cobbina et al. 2015).

Incidences of factories discharging effluent directly into the Ngong River have been reported (City Council of Nairobi 2013).

Substantial quantities of Zn, Pb, and As were recorded downstream of the Ngong River in the industrial area. Zn is an important component in alloy production while As is used in wood preservation and pesticide manufacturing (Tangahu et al. 2011; Water Environment and APHA 1999). Tanneries located around the Chokaa area may have discharged considerable quantities of Cr; 245 μg/L Cr was detected at the Nairobi-Ngong River confluence.

Previous study in China concurs with the present study by showing Cr and Zn may emanate from industrial wastewater (Yao et al. 2014; Zhang et al. 2010).

Encroachment of the riparian zone for farming was rampant (City Council of Nairobi 2013). Destruction of riparian vegetation may have significantly hampered phytoremediation. NO_3_^−^ which is an important component in CAN and NPK fertilizer formulation appeared to have a high concentration in areas the riparian zone had been encroached. NO_3_^−^ is highly soluble and easily leached and may seep into river water after plant fertilization (World Health Organization 2007).

In the current study, *E. crassipes*, floating macrophyte, was found to uptake NO_3_^−^. Fourteen falls water sampling site that was predominantly covered with *E. crassipes* recorded 104 μg/L NO_3_^−^, in the rainy season, compared to the next sampling site, upstream of the river, which had 2382 μg/L. Absence of macrophytes between Fourteen falls and adjacent macrophyte identification site (upstream of the river) underscored the role played by *E. crassipes*. High NO_3_^−^ in the aquatic ecosystem is associated with eutrophication (Chu and Rienzo 2012). Eutrophication may inhibit macrophyte growth and consequently result in wetland stress (Wu et al. 2015). In most eutrophic ecosystems, P is often the limiting factor. In the present study, the Nairobi River average P concentration was 1.5 and 2 mL/L, in the rainy and dry season, respectively. Fresh water P concentration above 0.02 mL/L accelerates eutrophication (Sharpley et al. 2003). Efficient reduction of P by *E. crassipes* may suppress eutrophication and enhance colonization and diversity of macrophyte.

The Ondiri swamp, the source of the Nairobi River, recorded the least heavy metal contamination among sampled sites, inferring anthropogenic pollution downstream. However, the Ondiri swamp P concentration was relatively high, 1591 μg/L, in the rainy season, and may be attributed to the presence of high organic matter content observed (Riemersma et al. 2006).

## Conclusion

The Nairobi River heavy metal and nutrient concentration was observed to have spatial variation. This variation may have been due to point sources such as industrial effluent and dumping of solid waste in the river water. Heavy metal and nutrient concentration in a river may be influenced by weather pattern. Wet weather may dilute pollutant concentration while hot and dry weather may result in high concentration. This study was done in October, 2015 and June, 2016. While June was hot and dry, October was wet and humid. Higher pollutant concentration was detected in 2016 compared to 2015. Seasonal variation experienced was due to the prevailing weather.

The Nairobi River heavy metal concentration was relatively high in Mn, Fe, and Zn compared to rivers in other countries, Table 2.

Mn, Fe, Cr, and Pb concentration exceeded the WHO permissible limit. This may be a concern bearing in mind the Nairobi River drains its water into the Athi River. The Athi River water is used downstream for domestic and irrigation purposes. High Mn concentration has been associated with low IQ and may damage the nervous system (Khan et al. 2012; Water Research Australia 2013). Besides natural sources, some Mn, mostly from potassium permanganate used as a disinfectant, may have originated from sewerage contamination. Cr and Pb are extremely toxic and carcinogenic, and their high concentration in the Nairobi River water is alarming. Their pollution was from point source. Pb was high at the Dandora dumping site while Cr was high at the Chokaa, Eastern By-pass, and Kalimoni sites. Cr pollution at the Chokaa site may be from tannery while peeling of chrome-plated motor bikes (which were being cleaned in the river) in the other two sites may be the main source of pollution. Fe, mostly from andisol soils in the study area, has been found to cause malignant tumor (Khan et al. 2013a, 2013b)

The Nairobi River’s average P concentration was 1.5 and 2 mL/L in the rainy and dry seasons, respectively. Concentration of more than 0.01 mL/L P in all sites visited may cause eutrophication and reduce water quality (Volterra et al. 2002). However, the presence of bioaccumulator macrophytes and impoundment may be facilitating self-purification of the Nairobi River as observed in Fourteen falls and previous study (Baseline Report 2012).

The Ondiri swamp, source of the Nairobi River, recorded 15.3 and 1590.8 μg/L for NO_3_^−^ and P, respectively, with no detectable heavy metal in October, suggesting heavy metal pollution downstream may be principally anthropogenic.

## Recommendation

1. Presence of effective macrophytes that can efficiently bioaccumulate detected pollutants is significantly important. Cr, Pb, Fe, and Mn that were found to exceed the WHO permissible limit for drinking water can be phytoremediated by *C. articulatus* (Farrag and Manal 2012). *C. articulatus* was observed in the three study sites. Consequently, riparian vegetation that has been cleared for agriculture should be restored to nurture macrophyte growth and enhance phytoremediation.
2. High P that was recorded may cause eutrophication and destroy the aesthetic value of the Nairobi River besides inhibiting optimum growth of macrophytes (Wu et al. 2015). *E. crassipes* though an invasive species was noted to considerably uptake P in the current study. Its presence may curb eutrophication and facilitate growth of other beneficial macrophytes.
3. Constructed wetlands have proved effective in Cr remediation in contaminated water (Vymazal and Brezinova 2014). Tanneries located along the Nairobi River tributaries should be encouraged to build constructed wetlands to remove Cr in their wastewater before discharging into the river.
4. Suitable solid waste disposal, especially e-waste at the Dandora dumping site which is the main source of Pb polluting the Nairobi River should be prioritized. E-waste may be recycled. Leached heavy metals from improperly disposed garbage pollute water bodies (Ideriah et al. 2012).
5. Cleaning of motor bikes on river banks should be discouraged. Most motor bikes were chrome plated and some Cr was noted to be deposited in the water from peel off in the current study.
